# Spherical harmonic based noise rejection and neuronal sampling with multi-axis OPMs

**DOI:** 10.1016/j.neuroimage.2022.119338

**Published:** 2022-09

**Authors:** Tim M. Tierney, Stephanie Mellor, George C. O'Neill, Ryan C. Timms, Gareth R. Barnes

**Affiliations:** Wellcome Centre for Human Neuroimaging, UCL Queen Square Institute of Neurology, University College London, 12 Queen Square, London WC1N 3AR, UK

## Abstract

In this study we explore the interference rejection and spatial sampling properties of multi-axis Optically Pumped Magnetometer (OPM) data. We use both vector spherical harmonics and eigenspectra to quantify how well an array can separate neuronal signal from environmental interference while adequately sampling the entire cortex. We found that triaxial OPMs have superb noise rejection properties allowing for very high orders of interference (L=6) to be accounted for while minimally affecting the neural space (2dB attenuation for a 60-sensor triaxial system). We show that at least 11^th^ order (143 spatial degrees of freedom) irregular solid harmonics or 95 eigenvectors of the lead field are needed to model the neural space for OPM data (regardless of number of axes measured). This can be adequately sampled with 75–100 equidistant triaxial sensors (225–300 channels) or 200 equidistant radial channels. In other words, ordering the same number of channels in triaxial (rather than purely radial) configuration may give significant advantages not only in terms of external noise rejection but also by minimizing cost, weight and cross-talk.

## 1 Introduction

Optically Pumped Magnetometers (OPMs) and Superconducting Quantum Interference Devices (SQUIDS) both measure the brain's neuromagnetic field (Baillet, 2017; Xia et al., 2006). However, the measured signal differs in both its amplitude and spatial information content. These differences arise because OPMs can be placed directly on the scalp and therefore sample higher spatial frequencies of the brain's magnetic field at greater magnitude (Iivanainen et al., 2021). In theory, these higher spatial frequencies should result in better spatial resolution, but if their information is to be represented without any form of signal aliasing, more dense arrays or custom sensor layouts are required, in addition to lower noise and/or more signal (Ahonen et al., 1993; Beltrachini et al., 2021; Tierney et al., 2020; Vrba and Robinson, 2002).

One powerful approach to quantify the degree of higher spatial frequency content in MEG has been to use the Signal Space Separation (SSS) method (Taulu and Kajola, 2005). This approach has the added benefit that it can simultaneously model an array's ability to reject environmental interference. This is because SSS provides a model for both the neural data and environmental interference. The models for the neuronal data and the interference, respectively are the gradients of irregular and regular spherical harmonics. The irregular harmonics tend to zero at coordinates far from coordinate system's origin (outside the sensor array) and are thus useful for modelling magnetic fields coming from inside the sensor array (brain signals). The regular harmonics are then used for modeling magnetic fields that come from outside the array (interference).

An open question for OPM recordings is how many orders of spherical harmonics are required to model the environmental interference and the neuronal data. Higher orders of harmonics will inevitably explain more data but come at the cost of fewer free parameters. With regards to interference, our previous work has suggested that the limited sensor numbers in typical OPM arrays can result in the attenuation of neuronal signal if too high a spatial order of interference is selected (Tierney et al., 2021). Furthermore, it is worth considering whether the ability of OPMs to measure in more than one direction affects the selection of the spherical harmonic model order for the neuronal signal and magnetic interference. This is an important consideration as previous work on cryogenic systems has led to diverging approaches to system design. For example, work on (SQUID based) cryogenic systems has suggested that a two-layer, sensor array (comprising 400 sensors) measuring in more than one direction should achieve shielding factors greater than 60dB using SSS (Nurminen et al., 2010). It has been demonstrated empirically even adding a small number of tangential channels improves shielding factors (Nurminen et al., 2013). However, other authors have argued that optimal system design (for maximizing SNR) consists of 1st order radial gradiometers combined with 3^rd^ order synthetic gradiometers (Fife et al., 1999; Vrba and Robinson, 2002).

Optimal system design for OPM recordings is less clear as one has the added issues of optimizing for wearability and subject movement (Boto et al., 2018). For example, optically pumped gradiometers offer promising noise cancellation properties (Limes et al., 2020; Sheng et al., 2017), yet as larger sensor baselines are used, one may compromise wearability. The tradeoff is therefore to shorten the baseline (Nardelli et al., 2020) but this will result in lower depth sensitivity (Hämäläinen et al., 1993; Vrba and Robinson, 2002). Software approaches for magnetometers not reliant on (fixed) reference arrays such as beamformers (van Veen and Buckley, 1988), SSS (Taulu and Kajola, 2005) or Signal Space Projection (Uusitalo and Ilmoniemi, 1997) are thus quite attractive. Recent work on OPMs has suggested that beamformers have their interference control improved by multi-axis recordings (Brookes et al., 2021) allowing for movement in excess of 1 m to be made (Seymour et al., 2021). With these issues in mind, it is worth exploring to what extent a single layer of vector OPMs can separate brain signal from magnetic interference and how this interacts with sampling density.

There are many diverging viewpoints and methods to optimize neuronal sampling with OPMs. One could use different definitions of optimality or information content (Beltrachini et al., 2021; Iivanainen et al., 2021), including minimization of aliasing (Tierney et al., 2020) or seeking to minimize correlation between sources (Boto et al., 2016). One extensive exploration of radial oriented OPMs suggest that between 177 and 276 sensors are required for sufficient sampling (Iivanainen et al., 2021). The broad estimate of requisite number of sensors arises due to the use of different basis sets (eigenbasis and spatial frequency basis) for the estimation of the spatial degrees of freedom in the data. The SSS basis set can also provide an estimate of the spatial degrees of freedom in the data and we compare this estimate with the estimate from the eigenbasis.

Throughout this work we rely on the same spherical-harmonic basis sets as used in SSS to explore the issues raised. We chose to use these basis sets as they provide a unifying theoretical framework for both neuronal sampling and environmental interference rejection. All of its interference rejection properties can be derived theoretically once the geometry of the MEG array is known. It also allows one to provide an upper bound on the number of spatial degrees of freedom in OPM data. We can therefore theoretically explore the interactions of multi-axis recordings and varying sampling densities while modeling neural data and magnetic interference. We expect these results to be useful for those wishing to design OPM arrays for MEG experiments.

## 2 Theory

### 2.1 SSS as a model of brain signal and interference

SSS represents the magnetic field (H) as a linear combination of the gradients of spherical harmonics ($Y_{lm}\left(\theta ,\varphi \right)$) with coefficients $\alpha_{lm}$ and $\beta_{lm}$. The full formulation for the magnetic field in spherical coordinates (r,θ,φ) is

(1) $$H\left(r,\theta ,\varphi \right)=\;-\mu_{0}\;\sum_{l=0}^{\infty }\sum_{m=-l}^{l}\alpha_{lm}\nabla \left[\frac{Y_{lm}\left(\theta ,\varphi \right)}{r^{l+1}}\right]-\mu_{0}\;\sum_{l=0}^{\infty }\sum_{m=-l}^{l}\beta_{lm}\nabla \left[r^{l}Y_{lm}\left(\theta ,\varphi \right)\right],$$

where $\mu_{0}$ is the magnetic permeability of free space. The first term represents the neural space (with the gradient of irregular spherical harmonics) while the second term represents the interference space (with the gradient of regular spherical harmonics). When modelling MEG recordings, each set of basis functions is truncated to a maximum value of l. This value is chosen in order to represent sufficient variance in the signal or sufficiently model environmental interference. The number of columns in each basis set is $l_{max}^{2}+2l_{max}$, where $l_{max}$ is the order of the harmonic used. The spherical harmonic basis functions ($Y_{lm}\left(\theta ,\varphi \right)$) are complex functions. In the current study we replace them with real valued harmonics (Wikswo and Swinney, 1984), as in previous work (Mellor et al., 2021). In cartesian coordinates (x,y,z) these harmonics ($S_{l}^{m}$) are defined as follows:

(2) $$S_{l}^{m}=\left\{ \begin{array}{c} aP_{l}^{\left|m\right|}\left(\frac{z}{r}\right)\sin\left(\left|m\right|\text{atan}\frac{y}{x}\right),\;m<0\\ aP_{l}^{0}\left(\frac{z}{r}\right),\;m=0\\ aP_{l}^{m}\left(\frac{z}{r}\right)\cos\left(m\text{atan}\frac{y}{x}\right),\;m>0 \end{array}\right.$$

where the associated Legendre polynomial ($P_{l}^{m}$) has the following form:

(3) $$P_{l}^{m}\left(\frac{z}{r}\right)={\left(-1\right)}^{m}2^{l}{\left(1-\frac{z^{2}}{r^{2}}\right)}^{m/2}\sum_{k=m}^{l}\frac{k!}{\left(k-m\right)!}{\left(\frac{z}{r}\right)}^{k-m}\left(\begin{array}{c} l\\ k \end{array}\right)\left(\begin{array}{c} \frac{l+k-1}{2}\\ l \end{array}\right),$$

with

(4) $$r=\sqrt{x^{2}+y^{2}+z^{2}},$$

and

(5) $$a=\left\{ \begin{array}{c} \sqrt{\frac{2l+1}{4\pi }\;},\;m=0\\ {\left(-1\right)}^{m}\sqrt{\frac{2l+1}{2\pi }\frac{\left(l-\left|m\right|\right)!}{\left(l+\left|m\right|\right)!}\;}.\;m\neq 0 \end{array}\right.$$

For ease of notation we refer to the basis set representing the neural space and interference space as Aand B**,** respectively.

(6) $$A=\;\left(\nabla \frac{S_{l}^{m}}{r^{l+1}}\right)\cdot \hat{n},\;B=\left(\nabla r^{l}S_{l}^{m}\right)\cdot \hat{n}.$$

With $\;\hat{n}$ being the unit vector representing the sensor's sensitive axis and · represents the dot product. We provide an explicit form for A and B in Appendix A and code to create these harmonics is made publicly available at https://github.com/tierneytim/OPM/blob/master/spm_opm_vslm.m. These harmonics can be used to reject magnetic interference from the data in one of two ways. Firstly, both the matrices A and B can be modelled simultaneously. This can be achieved by multiplying the pseudoinverse of the column-wise concatenation of matrices A and B (${\left[A\;B\right]}^{+}$) by the data (Y) to estimate the spherical harmonic coefficients (c)

(7) $$c={\left[A\;B\right]}^{+}Y.$$

The data can then be projected on to the subspace ($Y_{A}$) of A by multiplying A by the coefficients pertaining to its harmonics ($c_{A}$)

(8) $$Y_{A}=\;Ac_{A}.$$

Alternatively, one can simply project the matrix B from the data. This can be done by multiplying a matrix M by the data to get a cleaned version of the data ($Y_{\mathrm{clean}}$)

(9) $$Y_{\mathrm{clean}}=\;\mathrm{MY},$$

Where M is defined as follows

(10) $$M=\;I-BB^{+}.$$

The first approach (Eq. (7)) is how one would remove interference while performing SSS (Taulu and Kajola, 2005) and the second approach (Eq. (9)) is more equivalent to how one would remove an interference topography using SSP (Uusitalo and Ilmoniemi, 1997). Importantly, SSP is normally based on a noise space defined by empty room recordings. Here the OPM sensor array may move with the subject and so we use the external noise basis from SSS to define the noise space on a sample by sample basis*.* For both approaches to produce useful results the brain space and interference space (modelled by B) need to have limited correlation. In the case of SSS, high correlation will cause the estimate of the coefficients to be unstable, whereas in the SSP-style approach brain signal will be attenuated (Tierney et al., 2021) . Either way, exploring the orthogonality of interference and neuronal signal is of interest. The limits on shielding factors provided by the projector defined in Eq. (10) are explored In Appendix B.

### 2.2 The orthogonality of interference and neuronal data

By truncating the regular solid harmonic at some order of l the matrix B has $l^{2}+2l$ basis vectors (columns) with as many rows as there are channels. For the model of the interference (B) to be practically useful, it needs to share minimal variance with the lead fields (L). This is not guaranteed for every array design. To asses this we measure the lead field variance attenuation when the interference term is regressed from the data. The variance attenuation for each brain area in decibels (dB) can be described as follows

(11) $$attenuation_{i}=10\log_{10}\frac{var\left(ML_{i}\right)}{var\left(L_{i}\right)},$$

where $var(L_{i})$ is defined as

(12) $$var\left(L_{i}\right)=\sum_{j=1}^{n}\frac{{\left(L_{ij}-\bar{L_{ij}}\right)}^{2}}{n-1}\;.$$

The indices i,j refer to the magnetic field produced by the $i^{th}\;$brain area (in this case a vertex on a cortical mesh) at the $j^{th}$ sensor for n sensors. $\bar{L_{ij}}$ refers to the arithmetic mean for the *i*th $i^{th}\;$brain area across sensors. Now we have a metric for every brain area that summarizes how much variance is lost for a given regular solid harmonic order of B. We can also calculate these metrics for any sensor array or number of measurement axes. This is crucial for establishing robustness of a given array design to environmental interference.

### 2.3 The order of harmonics required to model the neural space

As OPMs sample higher spatial frequencies one would assume they would require higher orders of harmonics in matrix A to fully model the neural data*.* By truncating the regular solid harmonic at some order of l the matrix A has $l^{2}+2l$ basis vectors (columns) with as many rows as there are channels. We arbitrarily describe the data as adequately modelled when 99% of signal variance is explained in greater than 95% of brain regions. Similarly, to Section 2.2 we can measure the variance explained ($VE_{i}$) as

(13) $$VE_{i}=\frac{var\left(NL_{i}\right)}{var\left(L_{i}\right)},$$

where

(14) $$N=AA^{+}.$$

The order of harmonics required to model the neuronal data is important as using sufficiently high order harmonics would allow us to bandlimit (in terms of the spatial frequencies represented by the harmonics) the OPM data. Any interference or noise outside this bandlimit would then be suppressed. It also allows us to come up with an upper bound for the number of independent samples in the neuronal data.

### 2.4 Assessing the efficiency of the neural model

The more efficiently a given model can represent the neural data the more interference can be rejected. An “efficient” model would have fewer parameters than there are measurements. As a benchmark to compare the use of solid harmonics to model the neural data, we also consider eigenvectors (V) of the lead field covariance (C) matrix to model the neural data.

(15) $$C=LL^{t}=V\Sigma V^{t},$$

where Σ represents the covariance matrix's eigenvalues. Comparing the basis set V and A in terms of their efficiency is of interest as algorithms such as DSSP (Cai et al., 2019; Sekihara et al., 2016) project the data onto the lead field eigenvectors. The software for MEG/EEG analysis, SPM, also performs this step as a preprocessing procedure for source reconstruction (Friston et al., 2008; Lopez et al., 2014). As V is an orthogonal matrix the projector (O) that projects the data onto this basis is simply

(16) $$O=VV^{t}.$$

We can then once again calculate the variance explained as follows

(17) $$VE_{i}=\frac{var\left(OL_{i}\right)}{var\left(L_{i}\right)}.$$

In summary, we have now derived two subspace definitions which can be used to model neuronal signal, one based on solid harmonic gradients (A, Eq. (6)) and one based exclusively on the MEG system lead-fields (matrix V Eq. (15)). If we use the exact same threshold for when we consider the brain adequately modelled (>99% signal power in >95% of brain regions) we can compare the relative efficiency of both models (V and A) in representing neural data. We can then explore how both these models change as a function of sampling density and number of measured axes. If real data is projected on either of these subspaces the random internal sensor noise will be reduced by$\sqrt{Nc/Nr}$, where Nc is the number of channels and Nr is the number of regressors in either subspace. This forms an effective spatial oversampling factor. See Supplementary Fig. 1 for an example of white noise projected on to both subspaces V and A and the resulting square root dependence.

### 2.5 Considerations for on-scalp sampling

We also consider the implications of the using spherical harmonic models of neural data in on-scalp sampling situations. As one places sensors closer to the brain the relative influence of different sensors may change based on their distance to the origin (of the sensor coordinate system). This is because as the harmonic orders get higher in the matrix A, the dependency on the distance to the origin increases. If every sensor has the same distance to the origin (spherical sampling) this does not matter, but if sensors are not equidistant to the origin some sensors may have excessive influence on the modelling. This process can be captured formally with the statistical concept of Leverage.

(18) $$\text{Leverage}=diag\left(AA^{+}\right).$$

Each Leverage value tells us how influential a given observation is on the model. More formally it is the rate of change of the model with respect to the data. We can make this measure relative by dividing it by its mean value. Now each leverage value tells us how influential a data point is relative to the average data point. It is computed as follows:

(19) $$\text{Leverage}\left(\text{relative}\right)=\text{Leverage}\frac{n}{p},$$

where n is the number of sensors and p is the number of parameters of the model.

### 2.6 The comparison of sensors with different noise floors

When there is no external magnetic interference the signal to noise ratio (SNR) is simply the ratio of some signal (signal) to the standard deviation of the internal sensor noise ($\sigma_{sensor}$)

(20) $$SNR=\frac{signal}{\sigma_{sensor}}\;.$$

In the presence of interference, the Signal to Noise in Interference Ratio (SNIR) is defined as the ratio of the brain signal (signal) to the square root of the sum of the internal sensor variance ($\sigma_{sensor}^{2}$) and the external interference variance ($\sigma_{interference}^{2}$)

(21) $$SNIR=\frac{signal}{\sqrt{\sigma_{sensor}^{2}+\sigma_{interference}^{2}}}\;.$$

To simplify calculations, we model the standard deviation of the interference ($\sigma_{interference}$) as being some multiple (a) of the internal sensor standard deviation ($\sigma_{sensor}$)

(22) $$SNIR=\frac{signal}{\sqrt{\sigma_{sensor}^{2}+a^{2}\sigma_{sensor}^{2}}}=\frac{signal}{\sigma_{sensor}\sqrt{1+a^{2}}}=\;\frac{SNR}{\sqrt{1+a^{2}}}\;.$$

If some interference topography is removed, as described in Eq. (9), we would expect some attenuation factor (Att), in the range of 0–1, to weaken the signal when the interference and brain space are not orthogonal. Simultaneously, the shielding factor (SF) provided by removing this topography would reduce the ratio of interference to noise (a). These effects can be captured as follows:

(23) $$SNIR=\frac{SNR\;\left(Att\right)}{\sqrt{1+{\left(\frac{a}{SF}\right)}^{2}}}\;.$$

This expression can be used to work out the ratios of SNIR for both a radial ($SNIR_{Rad}$) and triaxial system ($SNIR_{tri}$)

(24) $$\frac{SNIR_{Rad}}{SNIR_{tri}}=\;\frac{w\left(SNR_{tri}\right)\left(Att_{rad}\right)}{\sqrt{1+w^{2}{\left(\frac{a_{tri}}{SF}\right)}^{2}}}\;\frac{\sqrt{1+{\left(\frac{a_{tri}}{SF}\right)}^{2}}}{SNR_{tri}\;\left(Att_{tri}\right)}=w\;\frac{Att_{rad}}{Att_{tri}}\sqrt{\frac{{\left(SF\right)}^{2}+{\left(a_{tri}\right)}^{2}}{{\left(SF\right)}^{2}+w^{2}{\left(a_{tri}\right)}^{2}}}$$

The only difference between $SNIR_{Rad}$ and $SNIR_{tri}$ is the presence of the scaling factor (w) which accounts for the fact that a radial system will have a higher SNR and interference to noise ratio ($a_{tri}$) due to having a lower noise floor. With this expression we can evaluate when a triaxial sensor will outperform a radial sensor in terms of SNIR.

## 3 Methods

### 3.1 Lead field generation

OPMs were modelled as point magnetometers displaced 6.5 mm from the scalp. The mesh used to generate these lead-fields was the MNI canonical cortical mesh available in SPM12 with 8196 vertices. The separation between vertices is approximately 5 mm on average. The orientation of the source was defined by the surface normal of the cortical mesh at that location. The forward model was the Nolte single shell model (Nolte, 2003). The sensors were placed on the scalp surface with separations of 85 to 15 mm in steps of 5 mm. The sensor placement algorithm is described elsewhere (Tierney et al., 2020). For each level of sensor spacing we simulated single axis, dual axis and triaxial sensors, generating 3 lead-field matrices per sensor spacing. Throughout the manuscript when we use the word “sensor” we are referring to a device that can *potentially* make multiple measurements in orthogonal directions. The word “channel” refers to 1 of these measurements (60 single-axis sensors have 60 channels; 60 dual axis sensors have 120 channels and 60 triaxial sensors have 180 channels).

For dual axis sensing, the second axis was set orthogonal to the radial axis. In order to define this second axis we set third component of the radial unit normal (say component *r* from vector [*p,q,r*]) to zero then swapped the remaining two components and negated the first (to give [*-q,p,0*]). This means the dot product of this vector with the original is zero (*-pq+qp+0*). The resulting vector was then normalised to have unit magnitude. For triaxial measurements the third axis was defined by the cross product of the first two.

### 3.2 The orthogonality of interference and multi-axis OPM recordings

We generated the first three orders of the regular solid harmonics in cartesian coordinates. The lead field attenuation (as documented in Section 2.2) was then calculated for each order, at each level of spatial sampling for single, dual and triaxial recordings, respectively. We also calculated the lead field attenuation for orders L=1 to 12 for single, dual and triaxial systems comprising of 60 and 400 sensors. This second analysis is intended to compare the limits of interference rejection in a realistic wearable array to an ideal, but impractical, array.

### 3.3 Order of harmonics required to model the neural space

We generated the first 15 orders (up to 255 vectors) of the real irregular solid harmonics in cartesian coordinates for the densest sampling (15 mm separation, 424 sensors). The variance this basis set explained in the lead fields was calculated for single, dual and triaxial systems, as described in Section 2.3. For comparison we also calculated the variance explained for magnetometers displaced 24 mm from the scalp (to represent a cryogenic, SQUID based system). At this point we also estimate the impact of non-spherical sampling on the model of the neural data. We computed the leverage as described in Section 2.5 and compared the sensors’ influence on the neuronal model to its distance to the origin (of the sensor coordinate system) at different orders of spherical harmonics (L=1, L=6, L=12). For comparison we also calculated these same metrics on a sphere. For simplicity of presentation we only examine one sensitive axis.

### 3.4 Assessing the efficiency of the neural model

The efficiency of the model to represent the neural data (the number of basis vectors required) is compared against the eigenvectors of the lead field which also form a compact basis set for describing the neural space (Iivanainen et al., 2021). To assess how many eigenvectors are required to model the neural space we compute the variance explained as in Section 2.4. We do this for all steps of spatial sampling for, single, dual and triaxial measurements.

### 3.5 Assessing the SNIR of sensors with different noise floors

The expression for the ratio of SNIR of radial to triaxial sensors (Eq. (24)) is a function of the ratio of external interference to white noise, achievable software shielding factors (limited by sensor calibration), harmonic order of the interference, the separability of the interference from the brain signal as well as the relative white noise floors of the sensors. The triaxial sensor was assumed to have a noise floor that is 2.5 times higher than a radial sensor (https://quspin.com/products-qzfm/). The attenuation of brain signal was established following the procedure outlined in Sections 3.1 and 2.2, for orders L=1, L=2 and L=3. Shielding factors were varied between 1 and 40 decibels while the ratio interference to noise was varied between 0 and 20. Eq. (24) was then evaluated for these parameter values to determine when radial sensors began to outperform triaxial sensors.

### 3.6 Software

Software required to generate the vector spherical harmonics described in this paper is made freely available on the first author's GitHub page (https://github.com/tierneytim/OPM). The key function is spm_opm_vslm. Examples and tests can also be found on GitHub (https://github.com/tierneytim/OPM/blob/master/testScripts/testVSM.m).

## 4 Results

### 4.1 The orthogonality of Interference and multi-axis OPM recordings

**Fig. 1:**
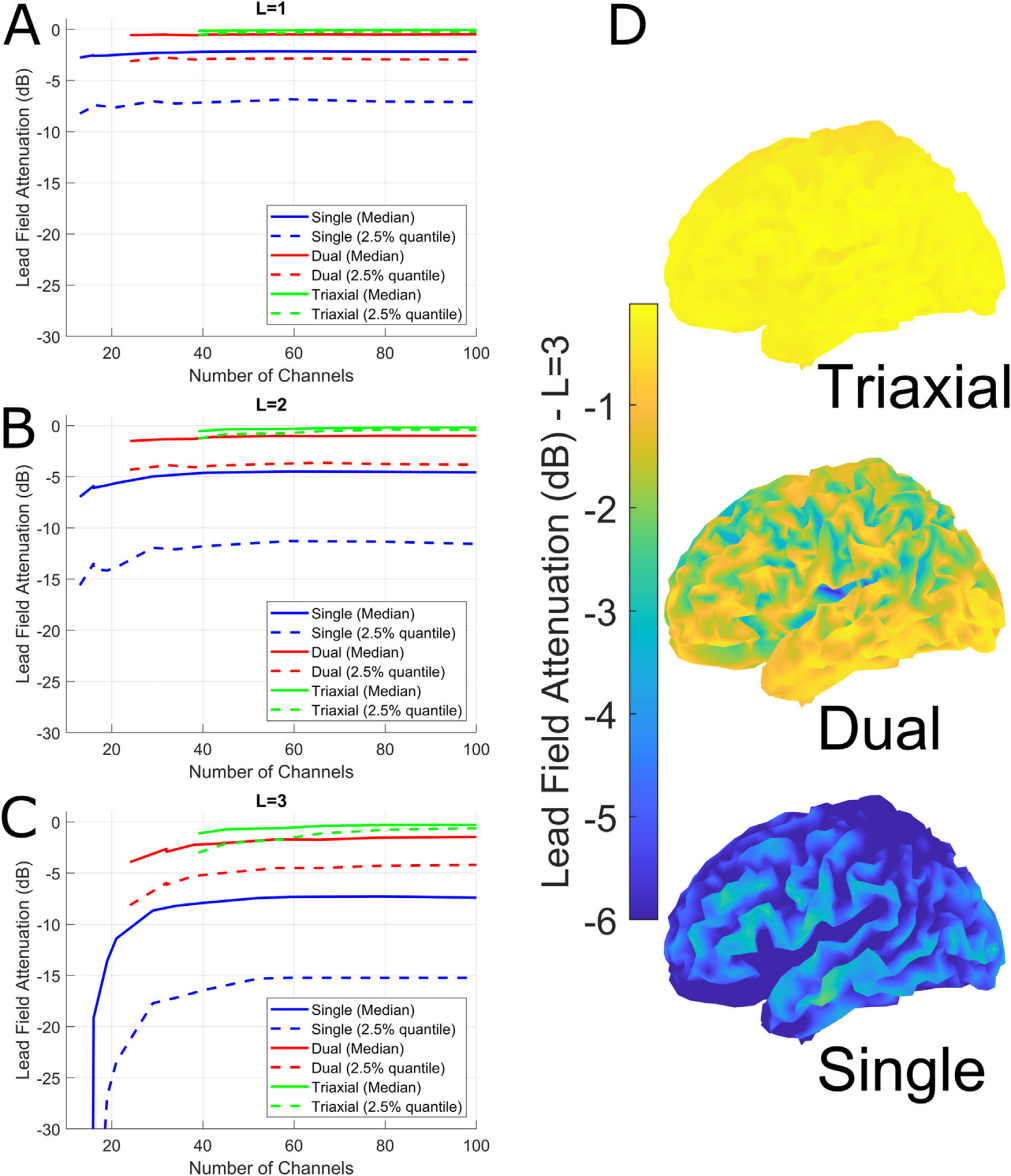
The orthogonality of interference and multi-axis OPM recordings. A, B and C show the expected lead field attenuation in decibels (y axis) when regressing regular solid harmonics of increasing order (L=1, L=2, L=3) from OPM data for single axis (blue), dual axis (red) and triaxial (green) channels. Solid lines show the median expected signal loss and dashed lines show the signal loss from the worst-affected top 2.5% of brain regions (worst case scenario). The x-axis indicates the number of channels. D shows the spatial distribution of the expected signal loss for L=3, for radial, dual-axis and triaxial channels. To summarize the figure, multi-axis recordings allow the removal of more complex environmental interference whilst preserving the neuronal signal. Importantly this is partly a factor of channel number but heavily determined by geometry; a given number of triaxial channels have less attenuation then the same number of single axis or dual axis channels (eg compare 60 tri-axial channels to 60 dual- or 60 single-axis channels).

Fig. 1 (A, B, C) shows the expected lead field attenuation when regressing regular solid harmonics of increasing order (L=1, L=2, L=3) from OPM data for single axis, dual axis and triaxial sensors. As expected, as the number of sensors is increased there is less risk of attenuating sensitivity to neuronal sources. Interestingly, all sensor types (triaxial, dual axis and single axis) converge rapidly to a channel-count-independent signal loss at all investigated orders. For greater than 90 channels the signal loss is lower than 1dB for channels configured triaxially even at high harmonic order (L=3). This is in contrast to single axis sensors which see greater than 15dB attenuation at this point. For L=3, This same information is represented spatially on the brain (Fig. 1D) for radial, dual and triaxial sensors. Ultimately, triaxial sensors allow for suppression of more spatially complex interference patterns with minimal risk of brain signal loss.

**Fig. 2:**
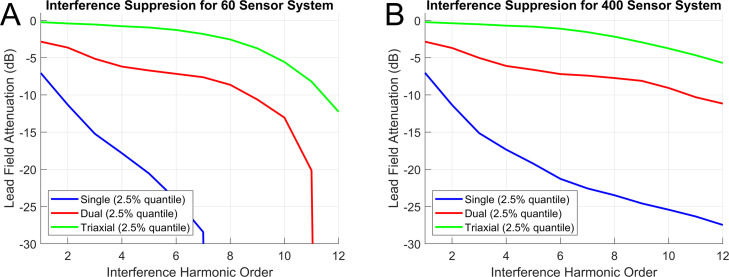
Statistical and physical limits of interference suppression for a 60 (A) and 400 sensor (B) OPM system. **A**. The 2.5% quantile lead field attenuation (decibels) for a 60-sensor system measuring single, dual and triaxial channels, respectively. In other words, for a triaxial system, one could remove ∼10 orders of external interference at the price of attenuating neuronal signals from 2.5% of the cortex by 5dBs. Triaxial systems offer clear advantages over dual and single axis systems in terms of minimal attenuation at high harmonic order. As the number of regressors ($order^{2}+2\;\times \;order$) approaches the number of channels the attenuation grows rapidly for all systems (representing the statistical limit of interference control). The same results are plotted in B for a 400-sensor system. In this case, the number of regressors never approaches the number of channels and we do not see the sudden attenuation of lead field variance at higher orders. In this case the lead field attenuation is representative of the physical limit of using higher order models.

In Fig. 2A and B we explore the statistical and physical limit of using high order models of interference on OPM data. Unsurprisingly, as the number of regressors ($order^{2}+2\;\times \;order$) approaches the number of channels the attenuation grows rapidly for all systems representing the statistical limit of interference control (Fig. 2A). In Fig. 2B when the number of channels is large relative to the number of regressors the lead field attenuation is determined by the spatial similarity of the magnetic fields generated by the brain and the interference space (representing a physical limit of using higher order models). However, one can see that for even a 60-sensor (180 triaxial channels) system the order of harmonics that one could remove from the data without exceeding 3dB of (neuronal space) attenuation for a triaxial system is greater than 6.

### 4.2 Order of harmonics required to model the neural space

**Fig. 3:**
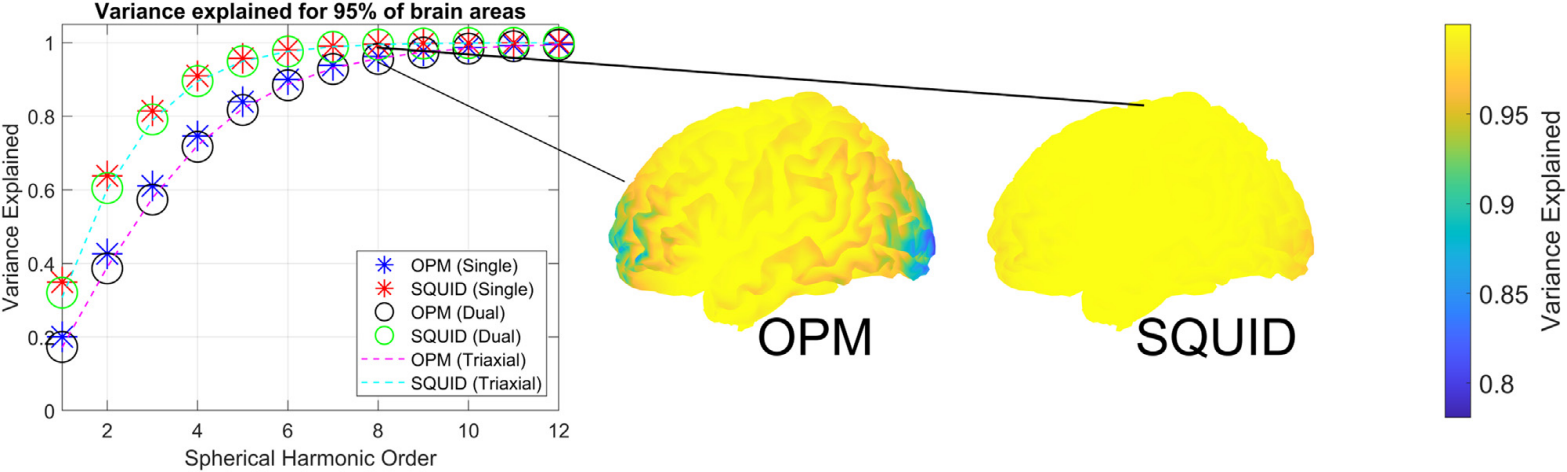
Order of irregular harmonics required to model OPM data due to brain activity. The y axis shows the variance explained for a given harmonic order (x axis) for OPMs and SQUIDS for single, dual and triaxial sensors (stars, circles and dashed lines repsectively). On the right we show the spatial profile of the variance explained across the brain for both OPMs and SQUIDS at L=8. Note that the loss of variance explained at the front and back of the brain when the (OPM) sensors are at the scalp surface. This is because these more distant sensors have less influence on the (spherical) neuronal model

Fig. 3 shows the order of irregular solid harmonics required to explain OPM data due to the brain (6.5mm scalp offset point magnetometers) and SQUID data (24mm scalp offset point magnetometers) for single, dual and triaxial systems. These simulations were performed using the simulated sensor arrays with 15mm sensor separation (424 radial channels, 848 dual axis channels and 1272 triaxial channels). Encouragingly, for the SQUID data (which only differs from the OPM data in scalp offset), the saturation of the harmonics (achieving 99% variance explained for 95% of brain regions) occurs at the same harmonic order (8) as in previous research (Taulu and Kajola, 2005). The OPM data saturates at L=11 implying there are at most 143 spatial degrees of freedom (see discussion for comparison with existing literature). Perhaps surprisingly, the results of Fig. 3 also imply that multi-axis measurements provide no new spatial information concerning the brain's activity when compared to single axis measurements (with large channel counts, 424 radial channels, 848 dual axis channels and 1272 triaxial channels). Essentially the dimensionality of the neural space does not increase as a function of multi axis measurement and the underling model (irregular solid harmonics) need not become more complex to explain multi-axis data. We will explore the same phenomenon in the next section with the eigenvectors of the lead field.

**Fig. 4:**
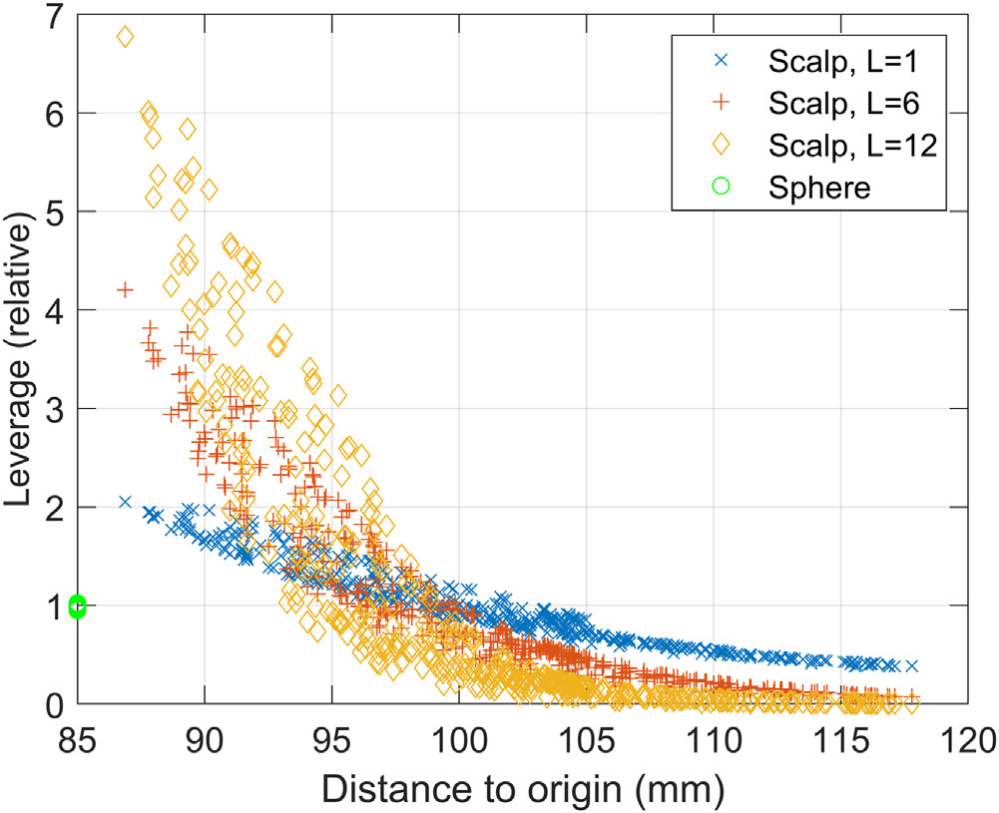
Sensor influence on irregular harmonic model of brain activity. Sensors displaced far from the origin of the coordinate system (x axis) have less relative leverage (y axis) than sensors closer to the coordinate system origin. This effect is stronger at higher orders of harmonics. Essentially, sensors displaced far from the origin have minimal impact on the model at high orders. This analysis is performed for both scalp-based sampling and sampling on a sphere. Note that although sampling on a sphere provides the most equitable impact of sensors on the model it is a special case where regular and irregular harmonics become highly correlated (perfectly correlated in the case of single axis radial or tangential channels (Nurminen et al., 2010)) rendering the separation of brain signal from interference highly challenging.

If we examine where the irregular solid harmonics poorly explain the brain data we note a spatial profile. As an example, at L=8 (Fig. 3 right) it would appear the harmonic model only explains 80% of the variance in areas at the front and back of the head in OPM data. This effect is largely explained by the sensors that are most distant from the origin of the coordinate system having the least influence on the model (Fig. 4). This phenomenon is more pronounced for data with higher spatial frequency content (such as OPM data) as higher harmonic orders (L=6, L=12) have many more highly influential sensors than at lower orders (L=1). Interestingly, in the case of spherical sampling, each sensor is equally influential on the model regardless of spatial frequency content.

### 4.3 Information content of multi-axis sensors at lower sampling densities

The results of the previous sections would suggest that while multi-axis recordings provide a much better model of magnetic interference over single-axis recordings (with comparable channel number) they provide no new spatial information about the neural signal. Essentially, the dimensionality of the neural space does not increase by using multi-axis recordings.

**Fig. 5:**
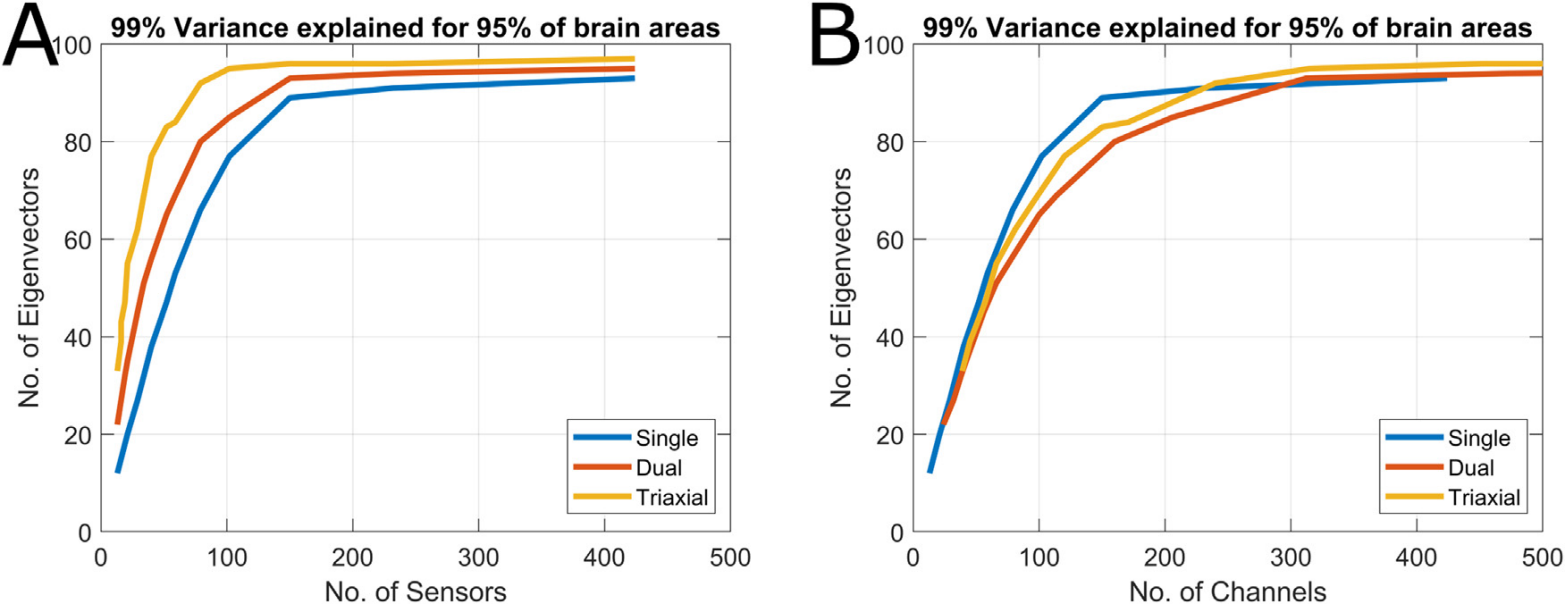
Eigenspectra of single, dual and triaxial OPM system. A. The figure shows the number of sensors (x-axis) vs the number of eigenvectors (y-axis) to achieve 99% variance explained in >95% of brain regions. When the number of eigenvectors stops changing as a function of sensor number, one has fully sampled the neural space. B shows the same information but the x axis is changed to channel count. The comparison of these two figures clearly shows that while triaxial systems allow for sampling the neuronal signal with lower sensor counts (A) radial-only systems can be constructed with lower channel counts to sample the same information (B).

We explore this result further by examining the eigenspetra of the leadfields. However, in this analysis we vary channel and sensor count to see how the dimensionality of the neural space changes. In Fig. 5, the number of eigenvectors required to explain 99% of variance in 95% of brain areas saturates at high sensor and channel count for all measurement types. This is similar to the results of the previous section demonstrating similar dimensionaliy (expressed in irregular solid harmonics) for large channel/sensor count measurments. The figure shows that there are diminishing returns (in terms of characterising neuronal signal) for triaxial systems after 75 sensors (225 channels). This is not the case for an equally distributed radial system which does not saturate until 150–200 sensors (or channels). The triaxial system completely saturates at 100 sensors (300 channels). However, if one were to control for channel count differences between sensor types then Fig. 5B shows that radial only systems can be constructed with lower channel counts to sample the same (neuronal) information.

### 4.4 SNIR comparison of sensors with different noise floors

**Fig. 6:**
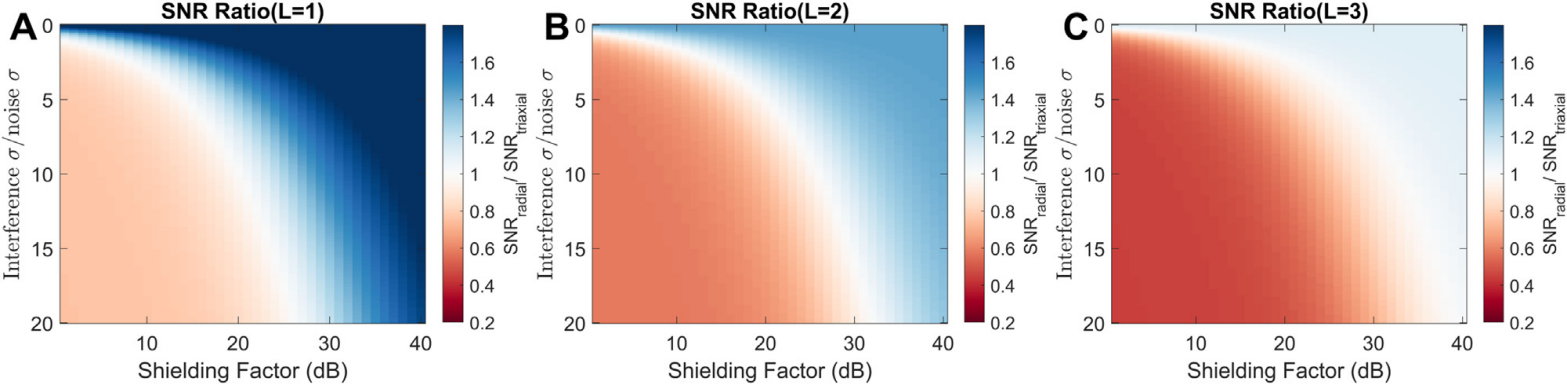
SNR ratio of purely radial vs. triaxially oriented channel arrays (figures derived from Eq. (24)). In A, B and C we show the ratios of SNR for radial to triaxial sensors for interference of harmonic order L=1, L=2, and L=3, respectively. The x-axis shows shielding factor in decibels while the y axis shows the ratio of the external interference to the internal white noise. The colours indicate whether triaxially (red) or radially oriented (blue) arrays have higher SNR. The shielding factor axis can also be seen as a proxy for sensor linearity as high shielding factors are only obtained with highly linear and well calibrated sensors. In summary the figures show that for low interference or for very high shielding factors (linearity) radial sensors are favoured. However, as the harmonic order (spatial complexity) or magnitude of the interference increases triaxial sensors become more favourable. These results assume that each triaxial channel has a noise floor that is 2.5 times higher than a radial sensor.

It is worth considering that measuring multiple axes also often incurs an increase in white noise. As such, a decision to choose between a radial or triaxial system will depend on a number of variables such as levels of interference, achievable shielding factors with postprocessing and the separability of interference and brain signal. As we have already established the separability of brain signal and interference in Section 4.1, we can combine this information with the theoretical results in Eq. (24) to explore when one can obtain better SNR with a radial sensor over a triaxial sensor. For the results in Fig. 6 we assume the triaxial sensor has a noise floor that is 2.5 times higher than a radial sensor (https://quspin.com/products-qzfm/).

In Fig. 6 we show the ratios of SNR of radial to triaxially oriented channel arrays where the noise floor is assumed to be 2.5 times higher for the triaxial channels than the radial. We look at the dependence of this ratio on shielding factor and interference strength. The achievable shielding factor varies as a function of sensor linearity and is explored in Appendix B. The attenuation due to post-processing is taken directly from the results of Fig. 1. For low interference or for very high shielding factors (obtained by ensuring the sensors are well calibrated) radial channels are favoured. However, as the harmonic order (spatial complexity) or magnitude of the interference increases then triaxial channels become more favourable. As the internal white noise floors become more comparable between radial and triaxial measurements, triaxial sensing is more generally favoured over radial only sensing (Supplementary Fig. 3).

## 5 Discussion

By using vector spherical harmonics and eigen-spectra as a theoretical basis we have explored the interference rejection and spatial sampling properties of single, dual and triaxial OPM data. We found that triaxial OPMs have superb noise rejection properties allowing for very high orders of interference (L=6) to be accounted for while minimally affecting the neural space (2dB attenuation for a 60-sensor triaxial system). The neural space was efficiently modelled by both irregular solid harmonics (L=11, number of harmonics =143) and by the eigenvectors of the lead fields (number eigenvectors < 100). Furthermore, SNR was higher for triaxial systems (even with increased noise floor) in the presence of spatially complex or large levels of environmental interference. We now discuss the implications for system design of these findings.

Spherical harmonics are commonly used to model the brain and magnetic interference with the SSS method (Clarke et al., 2020; Nurminen et al., 2010; Taulu et al., 2005; Taulu and Simola, 2006). Our work here reveals that for OPM data the default parameters derived for SQUID data should change. In SQUID data the neural space is typically modelled with harmonics of order 8 and the interference is modelled with harmonic of order 3. For OPM data (due to the higher spatial frequencies of magnetic field at the scalp) the neural space should be modelled with at least harmonics of order 11 (143 regressors) regardless of the number of axes measured. Alternatively, one could project the data on to a subset of the eigenvectors of the lead field as they form a more compact basis for the brain space (< 100 regressors required to represent the neural signal). This projection step reduces sensor white noise by the factor $\sqrt{Nc/Nr}$, where Nc is the number of channels and Nr is the number of regressors in either subspace (See Supplementary Fig. 1). This processing step is applied in all SPM source reconstruction algorithms (Friston et al., 2008; Lopez et al., 2014) and as a first stage in preprocessing for the noise rejection algorithm DSSP (Cai et al., 2019; Sekihara et al., 2016).

When modelling interference, the appropriate harmonic order strongly depends on the number of measurement axes. In short, radial only designs will perform poorly even at low harmonic order but triaxial systems will perform well at very high orders. Importantly, this effect is not driven by differing number of channels between single, dual and triaxial systems. We can see clearly in Fig. 1 that regardless of how many radial channels are utilized, the lead field attenuation (i.e. the loss of neuronal signal) will always be greater than that due to 20 triaxial sensors (60 channels). We should note that here we use SSS to define the external noise harmonics and then regress these (in a manner similar to SSP) from the data (Eq. (9)). If one were to try and model both the neural and external noise space as done typically in SSS (Eq. (8)), an extra 143 degrees of freedom would be required for the process to be stable.

While it is useful to examine the orthogonality of neural signals and external interference from different array designs, the decision to record from multiple axes is affected by a number of other variables. As well as neural signal attenuation one must consider the magnitude of the external interference, internal sensor white noise, the achievable shielding factors (which will be limited by sensor calibration and linearity) and the spatial complexity of the interference. By considering all these factors in Fig. 6, we can conclude that for higher shielding factors (due to better calibration) and lower levels of interference, radial samplings with a lower white noise floor – as is the current case with QuSpin OPMs - will outperform triaxial samplings in terms of SNIR. For greater magnitude and more spatially complex interference, triaxial arrays perform better. In other words, the ability to reject more external interference outweighs the white-noise penalty incurred through the use of triaxial measurements. In this study we have used a conservative estimate of the white noise levels (triaxial arrays are 2.5 times noisier) but provide a more realistic level (1.5 times higher) in Supplementary Fig. 3 based on recent empirical data (Boto et al., 2022). The trends are similar but more favorable to triaxial measurements.

Considering spatial sampling, we have built upon previous work which has shown that custom arrays can be designed to optimally sample a particular brain region (Beltrachini et al., 2021; Iivanainen et al., 2021; Tierney et al., 2020). In the current work we show that 100 equidistant triaxial sensors are sufficient to model the whole brain. In fact, for purely neuronal measurements, there are diminishing returns in having more than 75 triaxial sensors (Fig. 5A). However, the triaxial arrangement is not as efficient as a purely radial array (Fig. 5B). Essentially, while the vector components of a triaxial system do add independent information, the (neuronal) information gain per channel is smaller than it is for a radial only design. However, in addition to the enhanced noise rejection properties of triaxial sensors, there are practical considerations. As the manufacturing of triaxial OPMs negligibly affects their weight and cost, it may be beneficial to design a sparser triaxial array than a dense single axis array to optimize subject comfort and array wearability. Furthermore, the average sensor spacings are increased from 22 mm (for single axis only) to 32 mm for triaxial designs. Increasing the distance between sensors by 50% will have the added benefit of reducing the effects of cross-talk (Nardelli et al., 2019) by a factor > 2.

With regards to limitations we have not explored the implications of important effects such as volume currents which can be larger for tangential measurements (Iivanainen et al., 2017) or the impact of different forward models (Stenroos et al., 2014; Stenroos and Sarvas, 2012). There also are many other algorithms used for interference suppression that are not considered here. We chose to use SSS as a basis because all of its interference rejection properties can be derived theoretically once the geometry of the MEG array is known. It also allows one to provide an upper bound on the number of spatial degrees of freedom or dimensionality of OPM data. Other data driven techniques such as ICA (Vigârio et al., 2000), tSSS (Taulu and Hari, 2009) or the canonical correlation step in DSSP (Cai et al., 2019) are more difficult to consider because they are data driven. However, a full consideration of the applicability of these techniques for suppressing interference in OPMs is warranted. As a final point we have also not considered whether the positions and orientations of dual axis sensors could be further optimized to have performance similar to triaxial systems. In the current work we have first simulated a radial sensor and then arbitrarily chosen the second axis. In principle the first axis need not be radial and the second could be optimized.

**Fig. B1:**
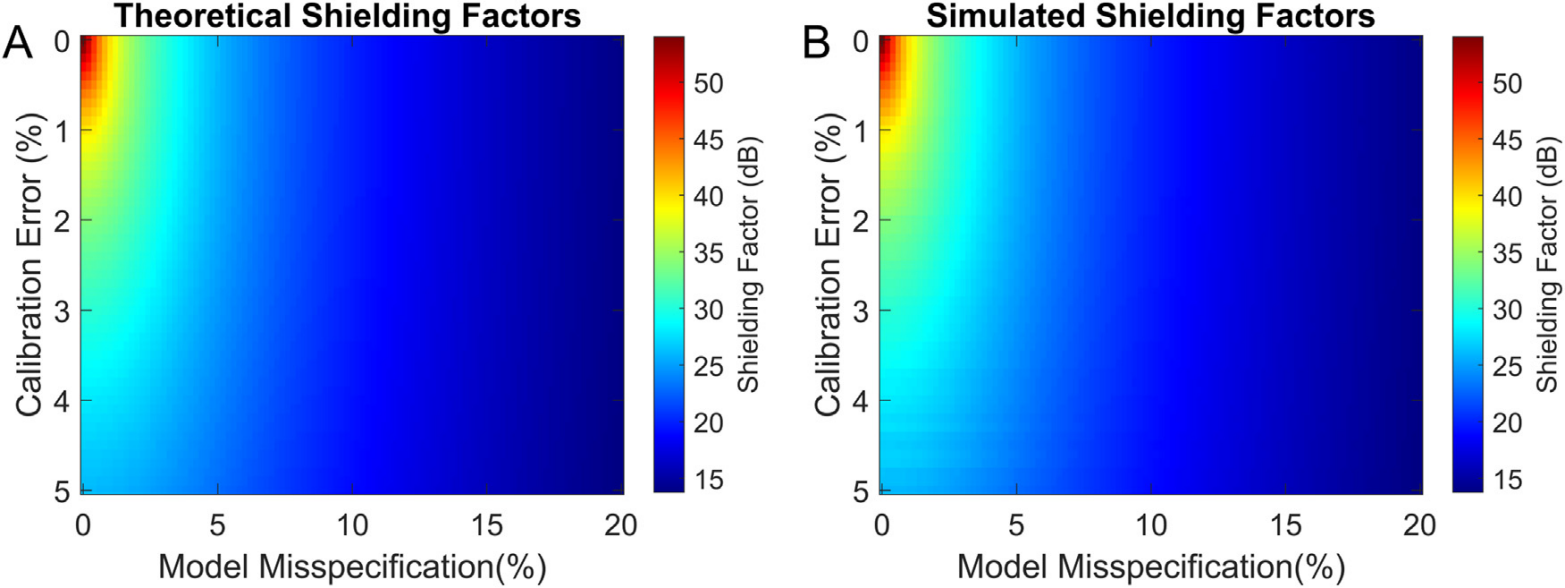
Theoretical and simulated shielding factors for regular harmonic projector. In A and B, the theoretical and simulated shielding factors (dB) are graphed as a function of calibration error (y-axis) and model misspecification (x-axis). The model misspecification term captures how well the projector removes interference (e.g. if a linear gradient accounted for 10% of interference and one only removed the homogenous terms, which accounted for the other 90% of interference, the model misspecification would be 10%). Ultimately the greatest shielding factors are only achieved when both the calibration error is low and the model is well specified.

With these limitations in mind, we have shown theoretically that triaxial OPM sensors are capable of separating signal from inference (with higher spatial frequency content) with minimal risk of attenuating brain signal when using regular solid harmonics. Furthermore, sparser arrays can be constructed with triaxial sensors than radial sensors simply because of the increased channel number. Triaxial designs also achieve higher SNR when interference is large or spatially complex. These findings all suggest that future systems based around triaxial arrays could allow for minimization of cost, weight and interference while maximizing the system's sensitivity to neural data in sparse arrays Fig. B1.

## Data and code availability

No data were acquired for this study. The software required to generate the vector spherical harmonics described in this paper is made freely available on the first author's GitHub page (https://github.com/tierneytim/OPM). The key function is spm_opm_vslm. Examples and tests can also be found on GitHub (https://github.com/tierneytim/OPM/blob/master/testScripts/testVSM.m).

## CRediT authorship contribution statement

**Tim M. Tierney:** Conceptualization, Methodology, Software, Writing – original draft. **Stephanie Mellor:** Conceptualization, Writing – original draft. **George C. O'Neill:** Software, Writing – original draft. **Ryan C. Timms:** Writing – original draft. **Gareth R. Barnes:** Writing – original draft, Methodology, Conceptualization.

## Appendix A Real, Cartesian, vector spherical harmonics

Here we provide explicit expressions for the matrices A and B introduced in Section 2.

$$A=\left(\nabla \frac{S_{l}^{m}}{r^{l+1}}\right)\cdot \hat{n}=\frac{\partial }{\partial x}\frac{S_{l}^{m}}{r^{l+1}}n_{x}+\frac{\partial }{\partial y}\frac{S_{l}^{m}}{r^{l+1}}n_{y}+\frac{\partial }{\partial z}\frac{S_{l}^{m}}{r^{l+1}}n_{z},$$

(A1) $$B=\left(\nabla r^{l}S_{l}^{m}\right)\cdot \hat{n}=\frac{\partial }{\partial x}r^{l}S_{l}^{m}n_{x}+\frac{\partial }{\partial y}r^{l}S_{l}^{m}n_{y}+\frac{\partial }{\partial z}r^{l}S_{l}^{m}n_{z},$$

where $\;n_{x},n_{y},\;n_{z}$ refer to the components of the unit vector ($\hat{n}$) representing the sensors sensitive axis. We introduce the variable t to refer to any of the axes, x,y,z and calculate the partial derivatives as below.

(A2) $$\frac{\partial }{\partial t}\frac{S_{l}^{m}}{r^{l+1}}=r^{-\left(l+1\right)}\frac{\partial }{\partial t}S_{l}^{m}-S_{l}^{m}\left(l+1\right)tr^{-\left(l+3\right)},$$

(A3) $$\frac{\partial }{\partial t}r^{l}S_{l}^{m}=r^{l}\frac{\partial }{\partial t}S_{l}^{m}+S_{l}^{m}ltr^{\left(l-2\right)}.$$

The only unknown in the above equation is $\frac{\partial }{\partial t}S_{l}^{m}$ which can be expressed as follows in Cartesian coordinates

(A4) $$\frac{\partial }{\partial x}S_{l}^{m}=\left\{ \begin{array}{cc} -aP_{l}^{\left|\left|m\right|\right|}\left(\frac{z}{r}\right)\left|m\right|\cos\left(\left|m\right|\text{atan}\frac{y}{x}\right)\frac{y}{x^{2}+y^{2}}+\sin\left(\left|m\right|\text{atan}\frac{y}{x}\right)a\frac{\partial }{\partial x}P_{l}^{\left|\left|m\right|\right|}\left(\frac{z}{r}\right), & m<0\\ a\frac{\partial }{\partial x}P_{l}^{m}\left(\frac{z}{r}\right), & m=0\\ aP_{l}^{m}\left(\frac{z}{r}\right)m\sin\left(m\text{atan}\frac{y}{x}\right)\frac{y}{x^{2}+y^{2}}\;+\cos\left(m\text{atan}\frac{y}{x}\right)a\frac{\partial }{\partial x}P_{l}^{m}\left(\frac{z}{r}\right), & m>0 \end{array}\right.$$

(A5) $$\frac{\partial }{\partial y}S_{l}^{m}=\left\{ \begin{array}{cc} -aP_{l}^{\left|\left|m\right|\right|}\left(\frac{z}{r}\right)\left|m\right|\cos\left(\left|m\right|\text{atan}\frac{y}{x}\right)\frac{x}{x^{2}+y^{2}}+\sin\left(\left|m\right|\text{atan}\frac{y}{x}\right)a\frac{\partial }{\partial y}P_{l}^{\left|m\right|}\left(\frac{z}{r}\right), & m<0\\ a\frac{\partial }{\partial y}P_{l}^{m}\left(\frac{z}{r}\right), & m=0\\ aP_{l}^{m}\left(\frac{z}{r}\right)m\sin\left(m\text{atan}\frac{y}{x}\right)\frac{x}{x^{2}+y^{2}}\;+\cos\left(m\text{atan}\frac{y}{x}\right)a\frac{\partial }{\partial y}P_{l}^{m}\left(\frac{z}{r}\right), & m>0 \end{array}\right.$$

(A6) $$\frac{\partial }{\partial z}S_{l}^{m}=\left\{ \begin{array}{c} \sin\left(\left|m\right|\text{atan}\frac{y}{x}\right)a\frac{\partial }{\partial z}P_{l}^{\left|m\right|}\left(\frac{z}{r}\right),\;m<0\\ a\frac{\partial }{\partial z}P_{l}^{m}\left(\frac{z}{r}\right),\;m=0\\ \cos\left(m\text{atan}\frac{y}{x}\right)a\frac{\partial }{\partial z}P_{l}^{m}\left(\frac{z}{r}\right),\;m>0 \end{array}\right.$$

Where

(A7) $$\frac{\partial }{\partial x}P_{l}^{m}\left(\frac{z}{r}\right)={\left(-1\right)}^{m}2^{l}\sum_{k=m}^{l}c\left(\frac{-xz^{k-m}\left(k-m\right)\left(\sqrt[{m}]{x^{2}+y^{2}}\right)+\left(xmz^{k-m+2}\right)\left(\sqrt[{m-2}]{x^{2}+y^{2}}\right)}{r^{2+k}}\right),$$

(A8) $$\frac{\partial }{\partial y}P_{l}^{m}\left(\frac{z}{r}\right)={\left(-1\right)}^{m}2^{l}\sum_{k=m}^{l}c\left(\frac{-yz^{k-m}\left(k-m\right)\left(\sqrt[{m}]{x^{2}+y^{2}}\right)+\left(ymz^{k-m+2}\right)\left(\sqrt[{m-2}]{x^{2}+y^{2}}\right)}{r^{2+k}}\right),$$

(A9) $$\frac{\partial }{\partial z}P_{l}^{m}\left(\frac{z}{r}\right)={\left(-1\right)}^{m}2^{l}\sum_{k=m}^{l}c\left(\frac{z^{k-m-1}\left(k-m\right)\sqrt[{m+2}]{x^{2}+y^{2}}+\left(-mz^{k-m+1}\right)\left(\sqrt[{m}]{x^{2}+y^{2}}\right)}{r^{2+k}}\right),$$

With c defined as follows

(A10) $$c=\frac{k!}{\left(k-m\right)!}\left(\begin{array}{c} l\\ k \end{array}\right)\left(\begin{array}{c} \frac{l+k-1}{2}\\ l \end{array}\right).$$

## Appendix B Theoretical and simulated shielding factors of the regular solid harmonic projector

We assume the data (Y) can be expressed as the sum of some interference term (I) and some random error term (ε).

(B1) Y=I+ε.

If the sensor is not appropriately calibrated the output deviates from its calibrated value and the updated signal can be described as follows

(B2) Y=I+CI+ε.

The calibration error matrix (C) is a diagonal matrix with non-zero elements being sampled from a normal distribution of mean zero and variance $\sigma_{C}^{2}$ ($N(0,\sigma_{C}^{2}$)). We note that this distribution may have a more complex form in real scenarios due to the presence of non-zero off-axis field (Borna et al, 2022). If one were to apply the projector described in Eq. (9). The sensor output would update as follows

(B3) MY=MI+MCI+Mε.

The shielding factor provided by the projector can now be described as the ratio of the variance of the data to the variance of the projected data.

(B4) $$SF=10\log_{10}\frac{var\left(Y\right)}{var\left(\mathrm{MY}\right)},$$

Where var is the symbol for variance. The variance of Y can be expressed in terms of the variance of the interference ($\sigma_{I}^{2}$), variance of the calibration error ($\sigma_{C}^{2}$), variance of the random noise ($\sigma_{\varepsilon }^{2}$) and the square of the arithmetic mean of the interference ($\mu_{I}^{2}$).

(B5) $$var\left(Y\right)=\sigma_{I}^{2}+\left(\sigma_{C}^{2}\right)\left(\sigma_{I}^{2}+\mu_{I}^{2}\right)+\sigma_{\varepsilon }^{2}.$$

The variance of the projected data (MY) can be approximated by noting that the variance of the unprojected calibration errors (CI) data forms an upper bound on the variance of the projected calibration errors (MCI).

(B6) $$var\left(\mathrm{MY}\right)<\;m\sigma_{I}^{2}+\left(\sigma_{C}^{2}\right)\left(\sigma_{I}^{2}+\mu_{I}^{2}\right)+\sigma_{\varepsilon }^{2}\frac{N_{c}-N_{r}}{N_{c}}..$$

The variance of the interference ($\sigma_{I}^{2}$) is now weighted by a value (m) between 0 and 1 representing how well the chosen model (or order) of interference matches the data. If m is low the model is well specified. If m is high the model is poorly specified. We also account for the reduction in the variance of the random error term ($\sigma_{\varepsilon }^{2}$) by multiplying the variance by the factor $\frac{N_{c}-N_{r}}{N_{c}}$, where $N_{r}$ is the number of regressors in the model and $N_{c}$ is the number of channels in the data. The theoretical shielding factors now achievable with the interference projector (M) can be described as follows

(B7) $$SF>\;10\text{lo}g_{10}\frac{\sigma_{I}^{2}+\left(\sigma_{C}^{2}\right)\left(\sigma_{I}^{2}+\mu_{I}^{2}\right)+\sigma_{\varepsilon }^{2}}{{\left(m\sigma_{I}\right)}^{2}+\left(\sigma_{C}^{2}\right)\left(\sigma_{I}^{2}+\mu_{I}^{2}\right)+\sigma_{\varepsilon }^{2}\frac{N_{c}-N_{r}}{N_{c}}}.$$

To verify these theoretical predictions, we simulate radial sensors on the scalp surface in the presence of 50pT interference (random coefficients of regular solid harmonic for L=1, L=2 and L=3 scaled to 50pT) and 100fT sensor white noise. We vary the calibration errors between 0 and 5% and the model misspecification term (m) between 0 and 20%. We vary this parameter by adding interference with variance ${\left(w\sigma_{I}\right)}^{2}$ to the data after the projector term (M) has been applied. These simulations were repeated 1000 times. The resulting shielding factors are plotted in Fig. B1 for the theoretical model and the simulated results. The agreement between theory and simulation is striking and implies that for the highest shielding factors (>40dB) the model misspecification needs to be low (<1%) as does the calibration error (<1%). However, for moderate calibration error (2%) and model misspecification (2%) shielding factors of 30dB are achievable. Encouragingly shielding factors of 20 dB are easily achieved even for considerable model misspecification and calibration errors (5%).

## Appendix C Supplementary materials

Image, application 1
