# Supplementary material for: Spherical harmonic based noise rejection and neuronal sampling with multi-axis OPMs
Source: Neuroimage. 2022 Sep;258:119338. doi: 10.1016/j.neuroimage.2022.119338 (PMC10509822; doi:10.1016/j.neuroimage.2022.119338)
Supplement: Supplementary file 1 [file mmc1.docx]

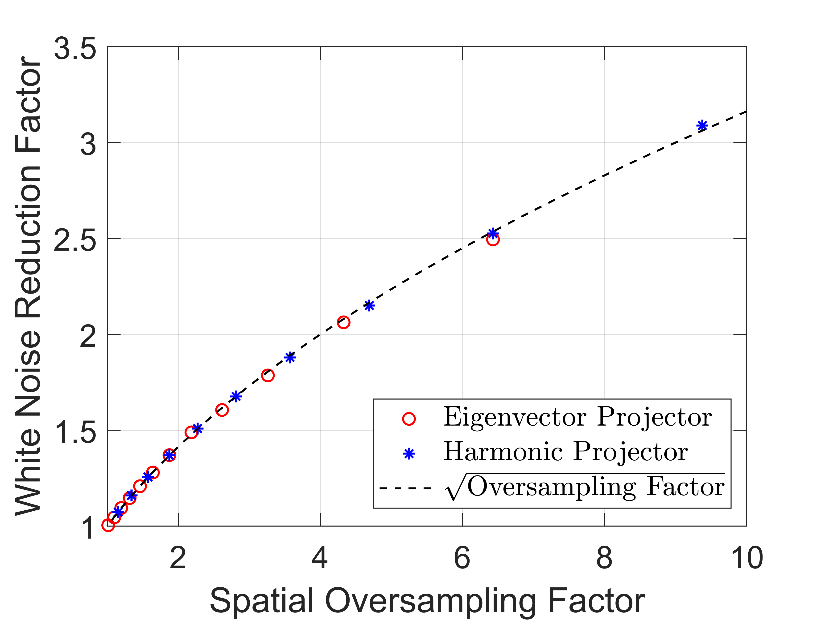


Figure S1. Spatial oversampling and white noise reduction. Spatial oversampling (x-axis) occurs when one projects a $Nc$ channel dataset onto a $Nr$ dimensional subspace. In this case, projecting white noise onto either the eigenvectors of the lead fields ($V$) or onto the irregular solid harmonics ($A$) results in a reduction in white noise (y-axis) by the factor $\sqrt{Nc/Nr}$. The lead fields and harmonics used here were created from a simulated array of 25 mm sampling (225 radial channels). The simulation was repeated 30 times with white noise and the results averaged. The blue stars represent different levels of compression for the harmonics while the red circles represent different levels of compression for the eigenvectors. Regardless of whether the harmonic or eigenvectors are used the white noise follows the same square root dependence on oversampling.


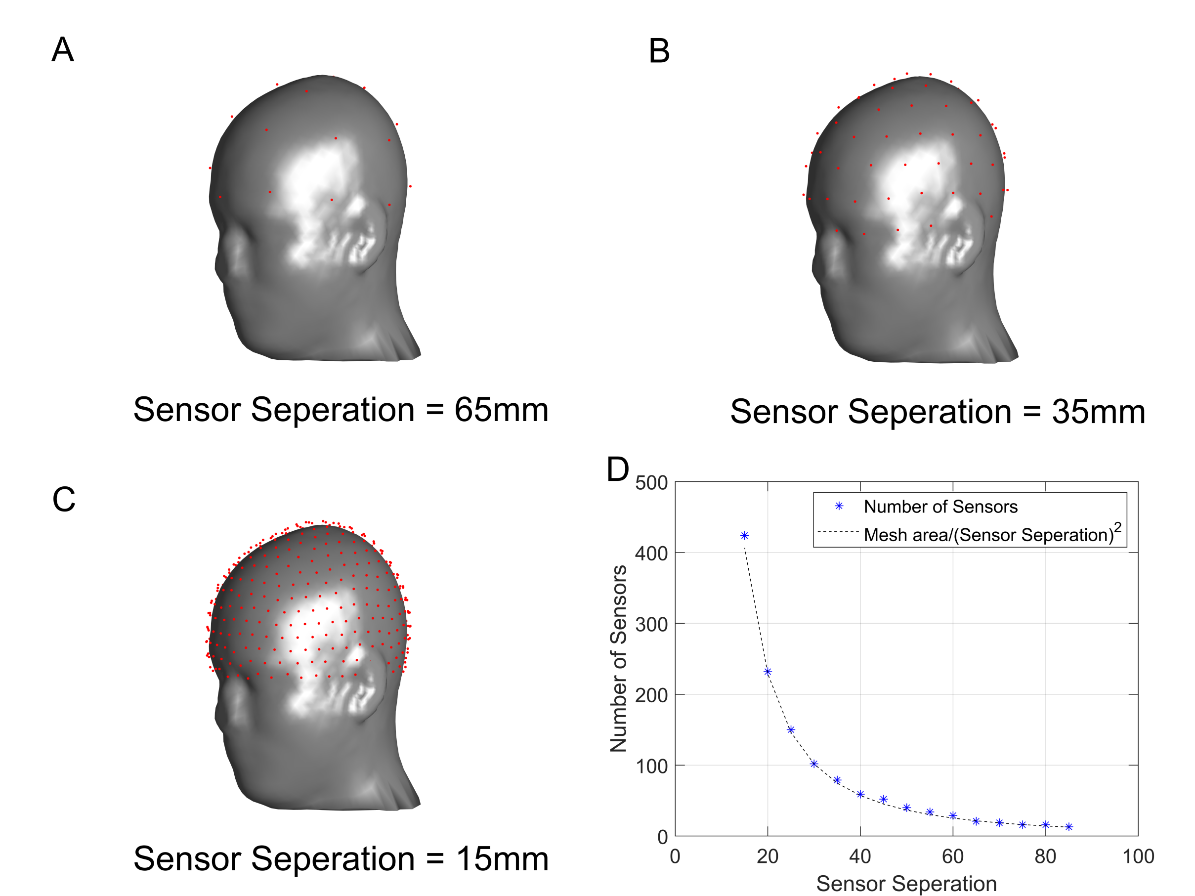


Figure S2. Exampled Sensor layouts and relationship between sensor separation and sensor number. A, B, and C show example sensor layouts for separation of 65mm, 35m and 15mm respectively on an MNI template scalp. Sensors are displaced roughly 6.5mm from the scalp and are represented by red dots. In D the relationship between sensor separation (x- axis) and sensor number (y-axis) is shown (blue stars). To good approximation the number of sensors placed by the algorithm is the scalp area divided by the square of the sensor separation (dashed black line).


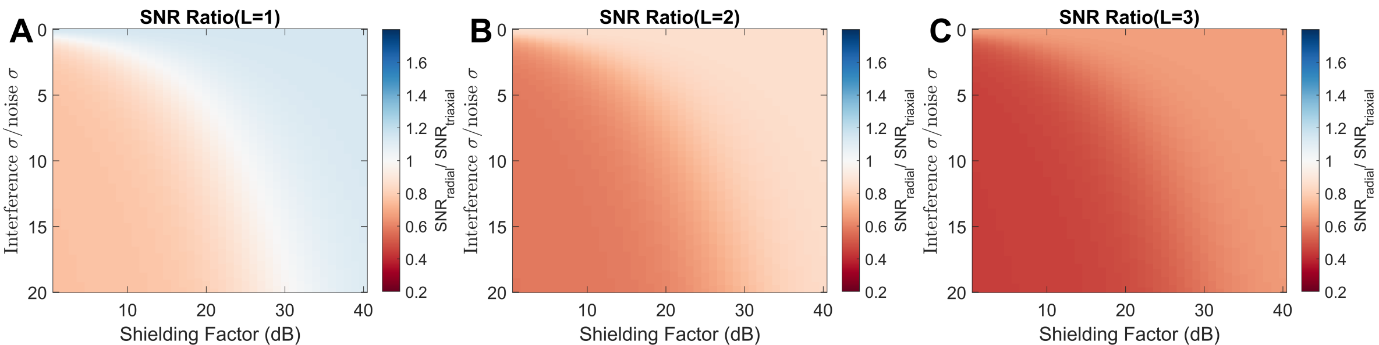


Figure S3. SNR ratio of radial to triaxial sensors. In A, B and C we show the ratios of SNR for radial to triaxial sensors for interference of harmonic order L=1, L=2, and L=3 respectively. The x-axis shows shielding factor in decibels while the y axis shows the ratio of the external interference to the internal white noise. The shielding factor axis can also be seen as a proxy for sensor linearity as high shielding factors are only obtained with highly linear and well calibrated sensors. In summary the figures show that for low interference or for very high shielding factors (linearity) radial sensors SNR improves. However, as the harmonic order (spatial complexity) or magnitude of the interference increases triaxial sensor become much more favourable. These results assume that the triaxial sensor has a noise floor that is 1.5 times higher than a radial sensor.
